# Structural Integrity of Steel Pipeline with Clusters of Corrosion Defects

**DOI:** 10.3390/ma14040852

**Published:** 2021-02-10

**Authors:** Maciej Witek

**Affiliations:** Gas Engineering Group, Warsaw University of Technology, 20 Nowowiejska St., 00-653 Warsaw, Poland; maciej.witek@pw.edu.pl

**Keywords:** steel pipeline, in-line inspection results, interacting corrosion defects, structural integrity

## Abstract

The main goal of this paper is to evaluate the burst pressure and structural integrity of a steel pipeline based on in-line inspection results, in respect to the grouping criteria of closely spaced volumetric surface features. In the study, special attention is paid to evaluation of data provided from the diagnostics using an axial excitation magnetic flux leakage technology in respect to multiple defects grouping. Standardized clustering rules were applied to the corrosion pits taken from an in-line inspection of the gas transmission pipeline. Basic rules of interaction of pipe wall metal losses are expressed in terms of longitudinal and circumferential spacing of the features in the colony. The effect of interactions of the detected anomalies on the tube residual strength evaluated according to the Det Norske Veritas Recommended practice was investigated in the current study. In the presented case, groups of closely-spaced defects behaved similarly as individual flaws with regard to their influence on burst pressure and pipeline failure probability.

## 1. Introduction

**Figure 1:**
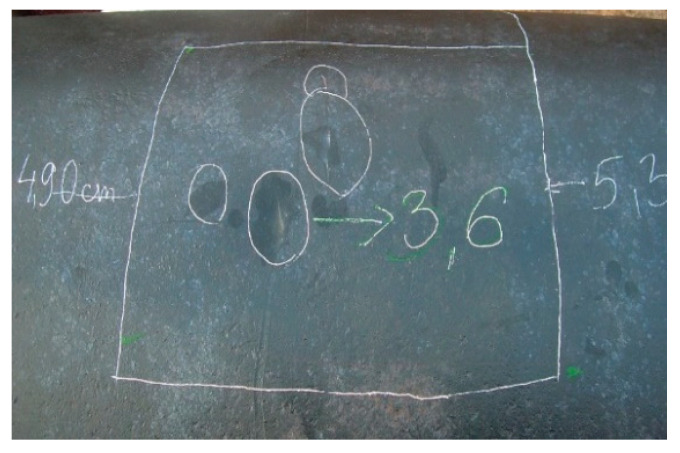
A photograph of corrosion colonies on the steel pipe surface. Author’s source.

**Figure 2:**
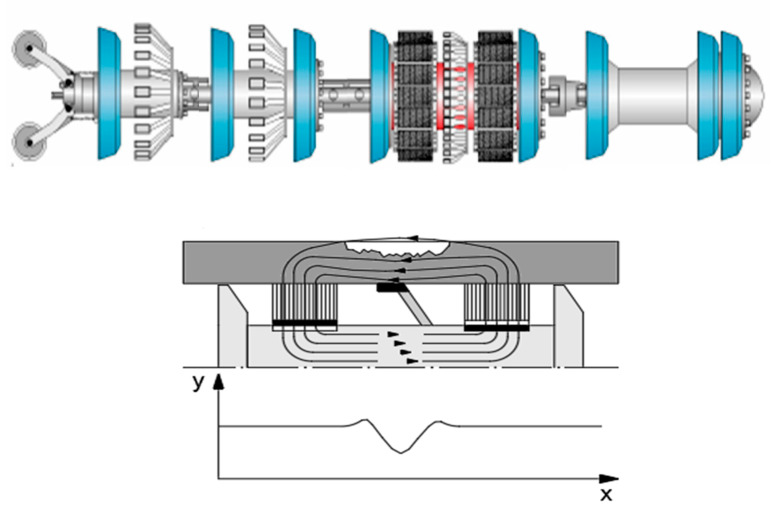
Axial excitation magnetic flux leakage inspection technology: a diagnostics tool (at the top) and a measurement principle (at the bottom). Author’s source.

Degradation of underground steel structures during their service lives leads to occurrence of volumetric surface defects and reduction of the tube wall thickness, as it is shown in Figure 1. The steel pipelines are buried almost on whole their length, and the properties of the soil are the most important factor of the corrosion; however, there are many other parameters such as an influence of the straight currents. A corrosion rate of high pressure steel pipelines needs to be controlled by their operators during the maintenance. Periodic in-line inspections (ILI), using an axial excitation magnetic flux leakage technology (Figure 2), are usually performed by gas transmission grid operators to detect and size the tube wall metal losses during the certain time intervals. If more than one diagnostic survey is performed at on a steel pipeline, so-called defect matching can be performed in order to evaluate the growth of the corrosion in the specific maintenance conditions [1]. Direct application of theoretical fracture mechanics methods to the assessment of the volumetric features provided from the in-line inspection is not appropriate due to uncertainty of the in-line inspection tool results highlighted by the author in [2]. The steel pipe wall burst was analysed by the author in [3]. However, a lot of studies deal with investigation of strength and structural integrity of steel pipelines with wall metal losses and longitudinally-oriented grooves similar to cracks using different methodologies, for instance, the latest publications applying a finite element method [4] and a linear elastic fracture failure mode [5]. A rupture pressure prediction model for steel tubes affected by the stray current corrosion based on artificial neural network was applied in [6].

Many research projects, in which the failure behaviour and assessment of cylindrical shells containing adjacent corrosion indications were investigated, have been carried out worldwide over the past 50 years. From those studies the conclusions are as follows. The interaction between each pair of metal losses within a group is governed by several parameters, namely, spacing, tube outside diameter, wall thickness, depth of anomalies, and shapes of defects. Among these factors, the distance between each pair of volumetric flaws is most significant. Rules of interaction of pipe wall metal losses are generally expressed in terms of longitudinal and circumferential spacing of the features in the cluster and are studied in many publications, for example [7]. The authors applied a finite element method to find new interaction rules of corrosion flaws for longitudinal and circumferential aligned metal losses as well as to compare these rules to the available grouping standards. However, the failure pressure of a cluster of closely spaced corrosion pits is generally smaller than the rupture pressures in the case when the defects are considered as isolated. This reduction in the corroded pipe strength occurs due to the possible interaction between the adjacent tube wall metal losses.

The influence of the results of analysis of the anomaly colonies on the rupture of the pipe subjected to the internal pressure was evaluated in the present study. There are two main subjects of interest in the investigation of closely spaced interacting corrosion defects. The first issue is the rules of grouping the metal losses, whereas the other one is prediction of the failure pressure of the pipe with adjacent defects. This research is focused on the assessment of tube wall volumetric surface flaws in respect to the criteria of interaction. In the present paper, DNV-RP-F101:2010 Det Norske Veritas Recommended practice [8] standard is applied to find defects which can be considered as clusters within the population of the external surface metal losses taken from the diagnostics data.

The mechanical reliability of the underground infrastructure within long-term operation, counted in decades, can be analysed as a stochastic process of random degradation of the structure elements. The algorithms calculating the probability of failure can be divided into methods based on functions of random parameters [9] and a theory of random variables which is applied in the current paper similarly as in [10,11]. There are plenty of publications which analyse the time dependent structural integrity relying on the stochastic models and on the results of the in-service diagnostics as well. Many publications, for example, [12], as well as the author’s work [13], calculate the failure probability considering defects as single isolated corrosion pits. However, a few publications focus on the interacting corrosion areas and, for this reason, this problem is the subject of the current research. The aim of the present study is to calculate the failure probability of the steel pipeline failure based on the in-line inspection data containing groups of indications. A limited number of publications focus on the multiple colonies of corrosion features and, for this reason, this issue is the subject of the current research. The following words: group, colony, cluster are synonyms and were used exchangingly in the text.

## 2. Clustering Rules for Tube Wall Metal Losses

There are two main subjects of interest in the investigation of the interacting pipe wall metal losses. The first issue are coincidence rules, whereas the other one is prediction of the failure pressure of a tube with adjacent colonies of defects. Section 2.1 presents the grouping criteria for the pipe wall volumetric anomalies detected by the axial excitation magnetic flux leakage in-line inspection tools. Section 2.2 contains the standardized assessment level 2 methodology for burst pressure calculations of a steel pipe with the interacting metal losses. 

### 2.1. Grouping Criteria for Volumetric Defect Colonies

According to the Benjamin Adilson’s classification presented in [14], there are three types of interactions of volumetric features caused by corrosion. Type 1 is a group of metal losses separated circumferentially; however, their individual profiles overlap when projected into the longitudinal plane through the wall thickness. Type 2 is found in colonies in which the flaws are or are not longitudinally aligned and their individual profiles do not overlap when projected onto the longitudinal plane through the wall thickness, i.e., their projected individual profiles are separated by the length of the full pipe wall-thickness. Type 3 is a combination of 2 above mentioned types. The described types are graphically shown in Det Norske Veritas Recommended practice [8].

The rules of interactions establish the limit value of the distance between two individual defects in the colony, beyond which the interaction is negligible. For the purpose of analysis of interactions, the longitudinal spacing *s_l_* and the circumferential distance *s_c_* of each metal loss in the group are usually verified. A majority of the currently available rules of clustering the corrosion flaws adopt expressions containing two following conditions to be met [8]:

(a) longitudinal spacing along the pipe axis is less than *s_Li_* ≤ (*s_l_*)_lim_
  (1) $$(s_{l}{)}_{\lim}=2.0\;\sqrt{Dt}$$
  and
(b) circumferential spacing *s_ci_* ≤ (*s_c_*)_lim_
  (2) $$(s_{c}{)}_{\lim}=p\;\sqrt{Dt}$$
  where:
  *s_li_*—longitudinal spacing of each defect in the colony, [mm];
  *s_ci_*—circumferential spacing of each anomaly in the group, [mm];
  *D*—tube outside diameter, [mm];
  *t*—pipe wall nominal thickness, [mm].

If the cluster is composed of more than two metal losses, the rules of interaction are applied to all possible pairs of adjacent defects within the group and the above mentioned criteria are verified. In the present study, the evaluation rules applied to the repeated in-line inspection data of gas transmission pipeline are presented.

### 2.2. Analytical Assessment of Interacting Defects

Det Norske Veritas Recommended practice Corroded pipelines level 2 methodology was developed for the calculation of the burst pressure of thin-walled cylindrical shells with interacting volumetric surface colonies and is applied in the current paper. The combined length of the interacting metal losses is calculated as follows [8]:

(3) $$l_{nm}=l_{m}+\overset{i=m-1}{\underset{i=n}{\sum }}\left(l_{i}+\;s_{i}\right)$$

where:

*l_i_*—axial length of each interacting metal loss from *n* to *m*, [mm];

*s_i_*—circumferential distance of each interacting metal loss from *n* to *m*, [mm].

*l_nm_*—combined length of defects in the longitudinal direction [mm].

The effective depth of the combined flaw formed from all *n* to *m* of the interacting metal losses is calculated as follows [8]:

(4) $$d_{nm}=\frac{\sum_{i=n}^{i=m}d_{i}l_{i}}{l_{nm}}$$

where:

*d_nm_*—effective depth of the metal loss combined from *n* to *m*, [mm];

*d*_i_—circumferential depth of each interacting metal loss from *n* to *m*, [mm];

The rupture pressure of the steel pipe with the combined corrosion colony formed from all *n* to *m* of the interacting metal losses is calculated as follows:

(5) $$P_{burst}=\;\frac{2tf_{u\;}\left(1-\frac{d_{nm}}{t}\right)}{\left(D-t\right)\left(1-\frac{d_{nm}}{tM_{nm}}\right)}$$

where:

*P_burst_*—burst pressure of the steel pipe with the combined defect formed from all *n* to *m*, [MPa];

*f_u_*—ultimate tensile strength of the steel, [MPa];

*M_nm_*—Folias factor, [–], corresponding to the combined length of defects in the longitudinal direction is expressed as:

(6) $$M_{nm}=\sqrt{1+0.31{\left(\frac{l_{nm}}{\sqrt{Dt}}\right)}^{2}}$$

## 3. In-Line Inspection Indications Clustering

**Figure 3:**
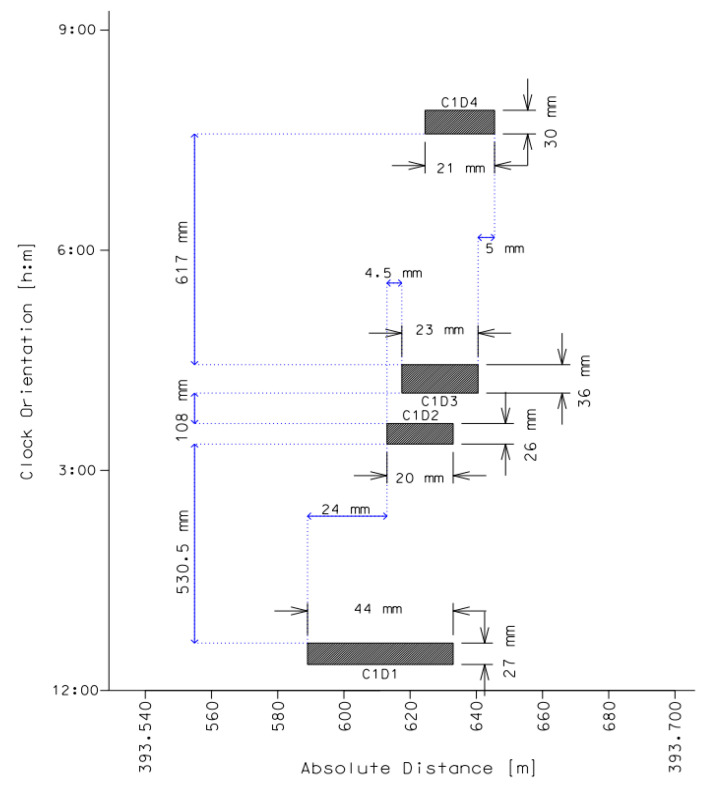
Graphical presentation of volumetric defects in colony 1. Source: Author’s analysis.

**Figure 4:**
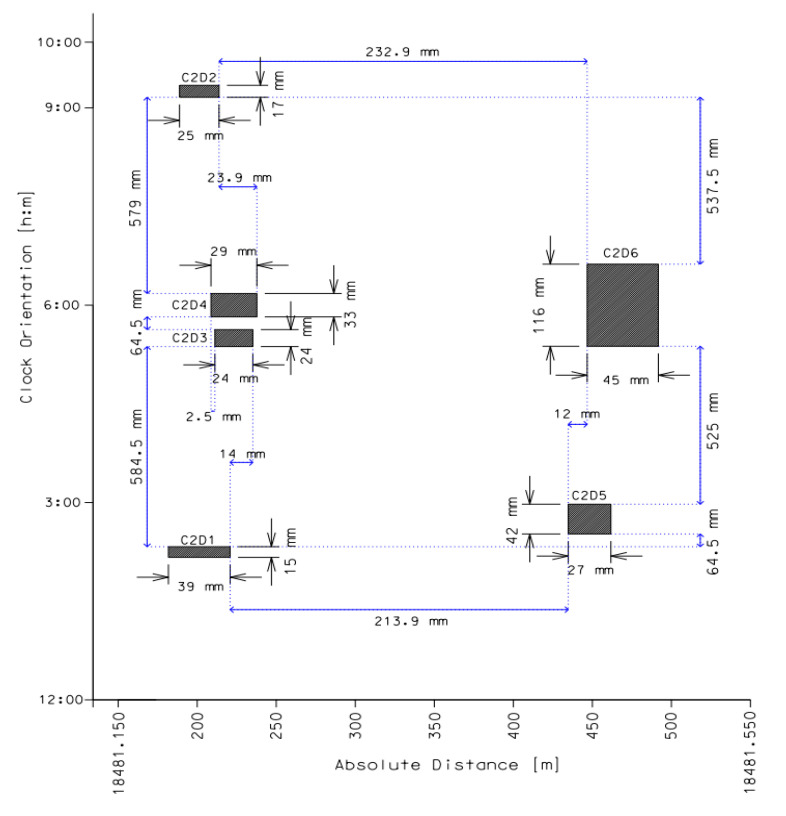
Graphical presentation of metal losses spacing in group 2. Source: Author’s analysis.

**Figure 5:**
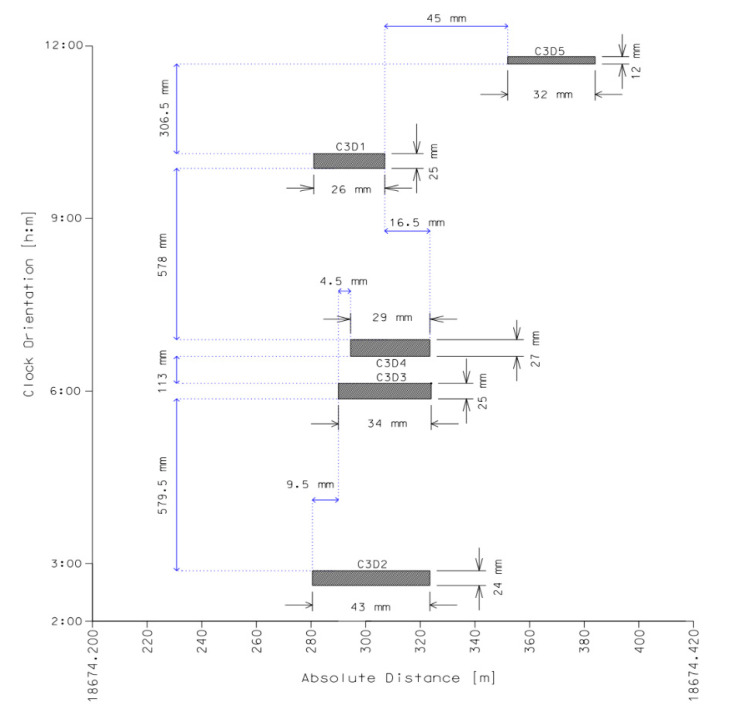
Graphical presentation of defects spacing in group 3. Source: Author’s analysis.

In order to illustrate the methodology for the interactions of defects described in Section 2, the clustering criteria were implemented to evaluate the possible material loss colonies along the studied gas transmission grid. The case study considers a 711 mm outer diameter cathodically protected pipeline of the total length of 147.267 km and the tube wall thickness of 10.5 mm. The maximum operating pressure value of all the pipeline sections is *MOP* = 5.5 MPa. The material used is equivalent to L360NE steel grade, according to EN-ISO 3183, with the following average parameters: ultimate tensile strength $\sigma_{U}\;$= 554.7 MPa, average yield stress $\sigma_{Y}\;$= 370 MPa and average elasticity modulus of steel *E* = 202 GPa. The pipe as well as the mechanical properties of the girth weld were confirmed by the destructive tests conducted on the steel coupons taken from the pipeline after 13 years of operation, directly after the first diagnostic [15]. Two in-line inspections were performed with the use of axial excitation magnetic flux leakage diagnostic tools. A period of time between the first and the second survey was 12 years. In this paper, all analyses are based on the real data for the external metal losses of the tube wall. Relying on the report of the first ILI, the number of 1347 external corrosion pits were found on the outside surface of the wall. Based on the data of the second ILI, the amount of 2838 external surface flaws were found. The number of 72 external closely spaced metal losses taken from the second inspection data were classified as groups for detailed considerations. For the research purpose, three of groups were described below in details and shown in Figure 3, Figure 4 and Figure 5. The limit of longitudinal spacing along the pipe axis in the studied case needs to be less than *s_L_* ≤ (*s_L_*)_lim_ = 176.9 mm and circumferential spacing *s_c_* ≤ (*s_c_*)_lim_ = 277.8 mm. Dimensions of the defects from group 1 are summarized in Table 1 and the graphical presentation of features spacing is shown in Figure 3. Description of defects in colonies are as follows: C1D1—cluster 1, defect 1.

Group 1 contains four flaws separated circumferentially; however, their individual profiles overlap when projected onto the longitudinal plane through the wall thickness (interaction type 1), as it is illustrated in Figure 3. Due to the limit of circumferential spacing for each pair of metal losses of the studied pipeline (*s_c_*)_lim_ = 271.5 mm, only two indications, C1D2 and C1D4, can be considered as interacting with the combined length of *l_nm_* = 27.5 mm and the maximum depth of 22% of the pipe wall thickness which corresponds to 2.42 mm. The effective depth of the external metal loss combined from C1D2 and C1D4 calculated according to Equation (4) is equal to *d_nm_* = 3.43 mm.

Dimensions of the defects from group 2 are summarized in Table 2. Cluster 2 contains six indications divided into two subgroups whose individual profiles overlap in the circumferential as well as in the longitudinal plane through the wall thickness (interaction type 1 and type 2), as it is shown in Figure 4. Due to the limit of circumferential spacing for the studied pipeline (*s_c_*)_lim_ = 271.5 mm and a value of longitudinal spacing along the pipe axis less than *s_L_* ≤ (*s_L_*)_lim_ = 172.8 mm of each pair, only two anomalies, C2D3 and C2D4, can be considered as interacting with the combined length of *l_nm_* = 29.0 mm and the maximum depth of 31% of wall thickness which corresponds to the value of 3.41 mm. The effective depth of the metal loss combined from C2D3 and C2D4 calculated according to Equation (4) is equal to *d_nm_* = 5.10 mm.

Dimensions of the metal losses from cluster 3 are summarized in Table 3. Colony 3 is the group of five anomalies separated circumferentially; however, four individual profiles overlap when projected onto the longitudinal plane through the wall thickness (interaction type 1), as it is illustrated in Figure 5. Due to the limit of circumferential spacing for the considered pipeline (*s_c_*)_lim_ = 271.5 mm and a value of longitudinal spacing along the pipe axis less than *s_L_*≤ (*s_L_*)_lim_ = 172.8 mm of each pair, only the two flaws C3D3 and C3D4 can be considered as interacting with the combined length of *l_nm_* = 33.5 mm and maximum depth of 22% of pipe wall thickness which corresponds to the value of 2.42 mm. The effective depth of the metal loss combined from C3D3 and C3D4 calculated according to Equation (4) is equal to *d_nm_* = 4.0 mm.

The results of the plastic burst pressure calculations according to Equations (5) and (6), in the three analysed cases of colonies of the volumetric defects, are summarized in Table 4.

From Table 4, it can be concluded that rupture pressure calculated according to Det Norske Veritas Recommended practice for every analysed group of flaws is in the difference range of 2 bar compared to the assessment of the individual features. The results of analytical calculation of burst pressure of the defected pipes, taking into consideration interactions of metal losses obtained from the in-line inspection, show a little influence of closely spaced corrosion flaws on the burst pressure of the studied case.

## 4. Probability of Pipeline Rupture

In order to estimate a failure probability in a long operation time, the limit state based on a pressure difference between the plastic collapse of the remaining pipe wall thickness and an expected value of the gas working pressure as a random variable were employed.

### 4.1. Calculation Methodology

The limit state function of the plastic collapse of the *j*-th pipeline section affected by a part-wall reduction caused by corrosion is expressed as:

(7) $$g(\vec{X)}\;=\;P_{fj}\;-\;OP_{max}$$

where:

$g(\vec{X)}$—limit state function of the tube wall plastic collapse;

$\vec{X}$—vector of random variables related to the pipeline segment;

$P_{fj}$—failure pressure of the j-th pipeline section affected by corrosion, [MPa];

$OP_{max}$—maximum operating pressure of the segment, [MPa].

The time-dependent theoretical failure pressure for a straight pipe with a part-wall volumetric surface defect is a function of the following variables:

(8) $$P_{fj}\left(T\right)=f(t,\;d,\;l,\;D,\;c_{d},\;\alpha ,\;c_{L},\;f_{u},\;T)$$

where:

*T*—time period, [year];

*c_L_*—axial corrosion rate for the defect length, [mm/year];

*c_d_*—proportionality coefficient of a power law function for the defect depth, [mm/year*^α^*];

*α*—exponential coefficient of a power law function for the flaw depth;

*f_u_*—ultimate tensile strength of the material used in design. [MPa].

Due to active corrosion of the pipe wall, the pipeline reliability decreases with time of the system operation. Increments of dimensions of metal losses during operation are described for the feature depth in radial direction with the following functions:

*d*(*T*) = *d_mean_*(T_0_) + *c_d_* ·*T^α^* (9)

and for the length of the longitudinally-oriented tube external surface features in the axial direction:

*L*(*T*) = *L*(T_0_) + *c_L_*·*T* (10)

Failure probability for a pipeline with corrosion grooving with time (*T*) can be calculated as follows:

(11) $$Pof_{j}\left(\vec{X},T\right)=P\left[g\left(\vec{X},T\right)\leq 0\right]$$

(12) $$P\left[g\left(\vec{X},T\right)\leq 0\right]\;=\;\overset{}{\underset{g\left(\vec{X},T\right)\leq 0}{\int }}f(x_{i},T)dx_{i}$$

where:

$Pof_{j}$—probability of failure of the j-th pipeline segment with an active corrosion defect, [1/year].

The failure occurs when $g\left(\vec{X},T\right)$ ≤ 0. For a specific time period, Monte Carlo numerical simulation is conducted by random generating of numbers for variables $P_{fj}$, with respect to statistical distribution of the input parameters specific for a segment. For each evaluation of limit state function (7), occurrence of $g\left(\vec{X},T\right)0$ is counted.

Probability of rupture of the j-th section of the pipeline, with the assumption of independence of *n* individual failures *Pof_jt_* of tubes connected in series, is calculated as

(13) $$Pof_{j}\left(\vec{X},T\right)\;=\;1\;-\;\overset{n}{\underset{t=1}{\prod }}(1\;-\;Pof_{jt}\left(\vec{X},T\right))$$

where:

*n*—number of pipe wall metal losses, [–].

For each external corrosion flaw, the total number of failure events *N_f_* is determined after Monte Carlo samples were generated and rupture probability of a single defect as a function of time can be obtained using the following Equation.

(14) $$Pof_{jt}\left(\vec{X},T\right)\;=\frac{N_{f}}{MC}$$

where:

*N_f_*—total number of failure events when $g\left(\vec{X},T\right)$ ≤ 0;

*MC*—total number of Monte Carlo trials at the specific time step for calculations of the *j*-th pipeline segment failure probability.

### 4.2. Input Data for Structural Integrity Evaluation

For the input parameters specified below, the pipe diameter and the wall thickness are modelled as random variables relying on the tube manufacturer’s certificates. The random variables listed below have been obtained from the two repeated diagnostics on the same pipeline. For ILI of oil and gas pipelines, it is a common practice to track the same anomaly in different inspections (i.e., so-called defect matching) based on the longitudinal and circumferential positions of the indication reported by ILI tools. Taken into consideration the clustering criteria, only six pairs of flaws from the total amount of 138 fully matched external corrosion pits were classified as interacting. Eventually, a number of 132 external metal losses are selected for structural reliability estimation taken into consideration the above mentioned interacting criteria.

**Figure 6:**
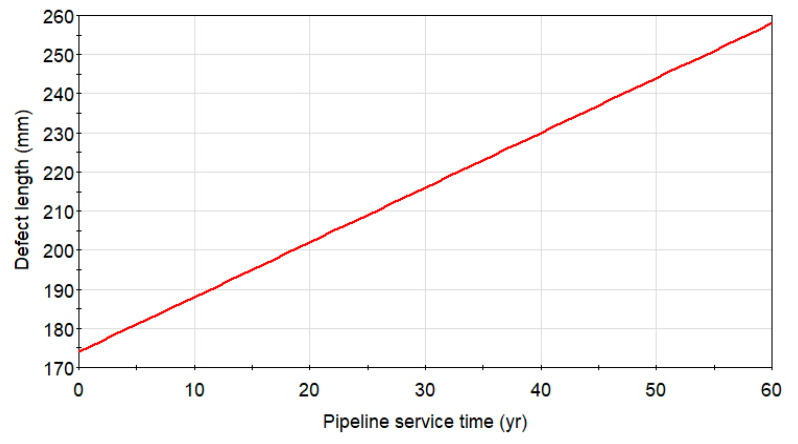
The linear length increment of the detected metal loss of the initial length l = 174 mm and a growth rate of 1.4 mm/year within the predicted pipeline service time. Source: Author’s analysis.

The external pipe surface corrosion coefficients for the cathodically protected underground structures are derived from the literature [16] and from the author’s other papers [13]. The reliability estimations of the pipeline were conducted with coefficients of corrosion growth rates according to Equations (9) and (10): *c_d_* = 0.164 mm, *α* = 0.78, *c_l_* = 1.4 mm/year. The linear length increment of the metal loss of the initial length *L* = 174 mm with a function of service time is presented in Figure 6.

The list of statistical distribution of input parameters for a structural integrity evaluation is presented in Table 5. Computations of failure probability were conducted with the use of an academic license of GoldSim software.

## 5. Pipeline Structural Reliability Considering Flaws Grouping

**Figure 7:**
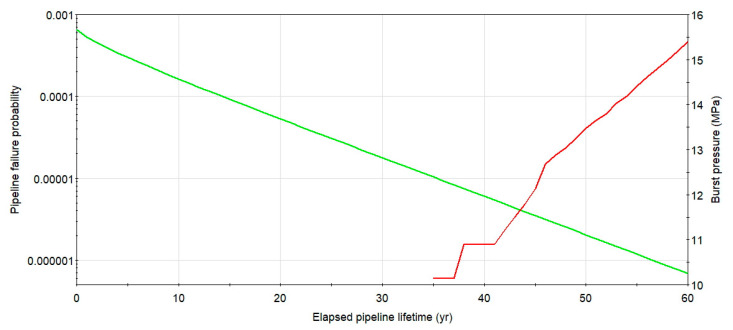
Change of rupture pressure and failure probability with time for X52 DN 700 *MOP* = 5.5 MPa pipeline with the longest detected defect and the initial depth of 12%. Source: Author’s analysis.

Integrity computations, taking into consideration the plastic collapse of the remaining wall thickness of the tube, were carried out relying on the in-line inspections data for the considered case. Due to a corrosion increment of the steel, the structural reliability decreases with time of the underground pipeline operation. The plot of the burst pressure (green line, right axis) and a probability of rupture logarithmic graph (red line, left axis) for the longest single metal loss of initial length *L* = 322 mm and maximum depth of 12% of the wall thickness, detected during the second ILI, as a function of service time for the considered case, is presented in Figure 7. The failure probability calculated for service time within 60 years starting from the second in-line inspection, even for non-repaired longest defect, is low and remain lower than a related code-based target value set for a pipeline safety class high as not higher than 10^−3^ per annum [8]. In the later years of maintenance, e.g., when the operation life of the studied pipeline is more than 40 years, a rate of the failure probability increase is strong, which means the rapid aging process of the steel underground structure.

**Figure 8:**
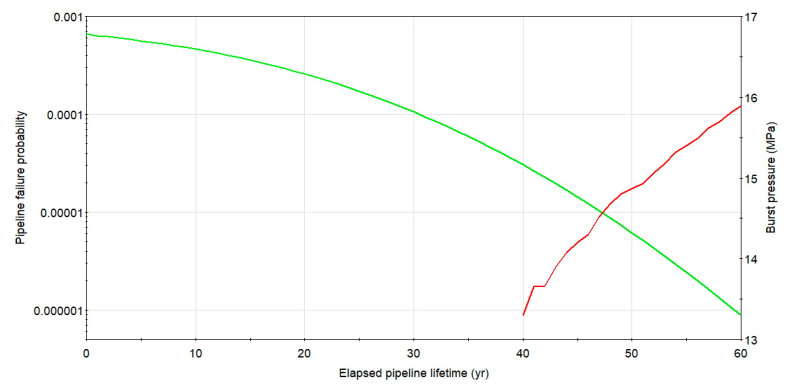
Change of rupture pressure and failure probability with time for X52 DN 700 *MOP* = 5.5 MPa pipeline with defect group1. Source: Author’s analysis.

The plot of the burst pressure and a probability of rupture logarithmic graph for cluster 1 with the combined length of *l_nm_* = 27.5 mm and the maximum depth of 22% of the wall thickness as a function of pipeline service time for the considered case is presented in Figure 8. The failure probability calculated for service time within 60 years starting from the second diagnostics, even for non-repaired defect colony 1, is low and remain lower than a related code-based target value set as not higher than 10^−3^ per annum. In the later years of pipeline service, exceeding 40 years, the failure probability increase is strong, showing the rapid aging effect of the steel buried structure.

**Figure 9:**
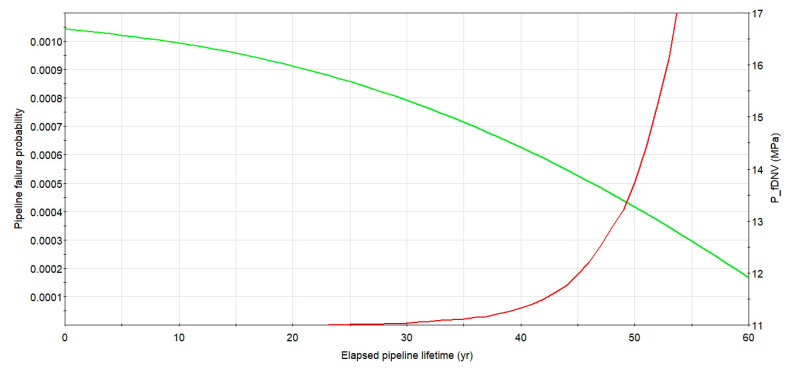
Change of burst pressure and failure probability with time for L360NE DN 700 *MOP* = 5.5 MPa pipeline with metal loss group 2. Source: Author’s analysis.

The graph of the burst pressure and a probability of rupture for flaw group 2 with the combined length of *l_nm_* = 29.0 mm and the maximum depth of 3.41 mm of the pipe wall thickness as a function of the operating time for the considered case is presented in Figure 9. The failure probability calculated for pipeline service in time, after 53 maintenance years, remains higher than a related code-based target value set for a safety class high as not exceeding 10^−3^ per annum.

**Figure 10:**
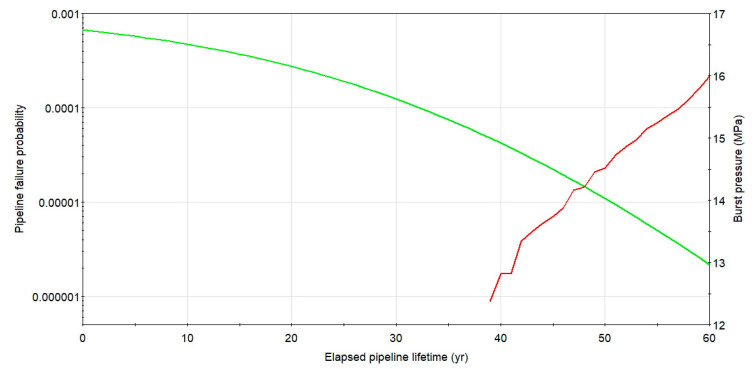
Change of burst pressure and failure probability with time X52 DN 700 *MOP* = 5.5 MPa pipeline with defect group 3. Source: Author’s analysis.

The plot of the burst pressure and a probability of rupture logarithmic graph for cluster 3 with the total length of *l_nm_* = 33.5 mm and the maximum depth of 22% of the wall thickness which corresponds to the value of 2.42 mm is shown in Figure 10. The probability of burst calculated for a pipeline operating period not more than 60 years, is low and remains lower than a related code-based target value set for a safety class high as not exceeding than 10^−3^ per annum. If the operation period of the studied buried pipeline is more than 40 years, a rate of the failure probability increase is strong, which means the rapid aging effect of the steel.

## 6. Conclusions

The calculation results of the burst pressure of the defected pipes relying on Det Norske Veritas Recommended practice Corroded pipelines, taking into consideration interactions of metal losses obtained from in-line inspections, show a little influence of closely spaced indications on the rupture pressure of the considered steel pipeline.

The probability of pipeline burst in the studied corrosion colonies cases do not differ significantly from the corresponding case when features are assessed as isolated. In the case considered in the present paper, grouping of closely spaced defects is almost consistent compared to the assessment of the individual flaws in respect of the burst pressure calculations and the probability estimations. The burst probability computations of the studied pipeline are independent of the results of corrosion grouping indications due to both small areas and the depth of the defects, which are the most important impact factors. The failure probability calculated for the pipeline service time within 50 years, starting from the second in-line inspection, even for non-repaired corrosion clusters, is low and remains lower than a related code-based target value set for a safety class high as not exceeding 10^−3^ per annum. In the later years of the studied pipeline operation, beyond 40 years, the structural integrity decrease is strong, which means the rapid aging degradation of the steel underground structure.

## Figures and Tables

**Table 1:** Anomaly dimensions in cluster 1. Source: Author’s analysis.

Defect №	Absolute Distance [m]	Relative Depth (d/t) [%]	Axial Length [mm]	Width [mm]	Clock Orientation [hour:min]
C1D1	393.611	17%	44	27	12:30
C1D2	393.623	22%	20	26	03:30
C1D3	393.629	25%	21	30	07:45
C1D4	393.635	20%	23	36	04:15

**Table 2:** Metal loss sizing in group 2. Source: Author’s analysis.

Defect №	Absolute Distance [m]	Relative Depth (d/t) [%]	Axial Length [mm]	Width [mm]	Clock Orientation [hour:min]
C2D1	18481.201	29%	39	15	02:15
C2D2	18481.201	25%	25	17	09:15
C2D3	18481.222	21%	24	24	05:30
C2D4	18481.222	31%	29	33	06:00
C2D5	18481.447	16%	27	42	02:45
C2D6	18481.468	19%	45	116	06:00

**Table 3:** Flaws dimensions in the colony 3. Source: Author’s analysis.

Defect №	Absolute Distance [m]	Relative Depth (d/t) [%]	Axial Length [mm]	Width [mm]	Clock Orientation [hour:min]
C3D1	18674.294	17%	26	25	10:00
C3D2	18674.302	22%	43	24	02:45
C3D3	18674.307	19%	34	25	06:00
C3D4	18674.309	22%	29	27	06:45
C3D5	18674.368	27%	32	12	11:45

**Table 4:** The results of the pipe wall plastic collapse calculated according to DNV-RP-F101 standard. Source: Author’s analysis.

Colony №	Defect №	Defect Depth [mm]	Axial Length [mm]	Burst Pressure [MPa]	Colony №
C1	C1D2	2.42	20.0	17.875	C1D2
	C1D4	2.20	23.0	17.868	C1D4
C2	C1D2 + C1D4	3.43	27.5	17.796	C1D2 + C1D4
	C2D3	2.31	24.0	17.861	C2D3
C3	C2D4	3.41	29.0	17.784	C2D4
	C2D3+ C2D4	5.10	29.0	17.666	C2D3 + C2D4

**Table 5:** Statistical distribution of input parameters for reliability evaluation. Source: Author’s analysis.

No.	Parameter	Unit	Mean Value	Uncertainty Coefficients	Distribution Type
1.	Yield tensile strength (f_y_)	MPa	370.6	StD[f_y_] = 12.2 COV[f_y_] = 3.3 %	Lognormal
2.	Ultimate tensile strength (f_u_)	MPa	554.7	StD[f_u_] = 19.4 COV[f_u_] = 3.5 %	Lognormal
3.	Pipe wall thickness (*t*)	mm	11.0	StD[*t*] = 0.5 COV[*t*] = 4.5 %	Normal
4.	Tube outside diameter (*D*)	mm	711.0	StD[*D*] = 20.3 COV[*D*] = 2.8 %	Normal
5.	Maximum operating pressure (*MOP*)	MPa	5.5	s = 0.3 COV[*MOP*] = 5.5 %	Gumbel
6.	Metal loss depth (*d*)	mm	defect specific	StD[*d*] = 0.6 COV[*d*] = 26.6 %	Normal
7.	Flaw length (*L*)	mm	defect specific	StD[*L*] = 34.6 COV[*L*] = 76.9 %	Lognormal
8.	Defect depth growth rate *d*(*T*) as a power law function with parameters *c_d_*, *α*.	mm/year	*c_d_* = 0.164, *α* = 0.78	Parameters *c_d_*, α deterministic	Parameters cd, α deterministic
9.	Metal loss length growth rate *L*(*T*) as a power law function with parameters *c_l_*.	mm/year	*c_l_* = 1.4	Parameter *c_l_* deterministic	Parameter cl deterministic

## Data Availability

The data presented in this study are available on request from the corresponding author.
